# Atypical Hemolytic Uremic Syndrome and Chronic Ulcerative Colitis Treated with Eculizumab

**DOI:** 10.9734/IJMPCR/2015/18771

**Published:** 2015-06-19

**Authors:** Tennille N. Webb, Heidi Griffiths, Yosuke Miyashita, Riha Bhatt, Ronald Jaffe, Michael Moritz, Johannes Hofer, Agnieszka Swiatecka-Urban

**Affiliations:** 1 Department of Pediatric Nephrology, Children's Hospital of Pittsburgh of UPMC, Pittsburgh, Pennsylvania, USA.; 2 Department of Pediatrics, Children's Hospital of Pittsburgh of UPMC, Pittsburgh, Pennsylvania, USA.; 3 Department of Gastroenterology, Children's Hospital of Pittsburgh, Pittsburgh, Pennsylvania; 4 Department of Pathology, Children's Hospital of Pittsburgh, Pittsburgh, Pennsylvania, USA.; 5 Department of Pediatrics, Innsbruck Medical University, Austria.; 6 Department of Clinical Biology and Physiology, University of Pittsburgh School of Medicine, One Children's Hospital Drive, 4401 Penn Avenue, Pittsburgh, Pennsylvania, USA.

**Keywords:** Hemolytic–uremic syndrome (HUS), thrombotic microangiopathy (TMA), ulcerative colitis (UC), inflammatory bowel disease (IBD), acute kidney injury (AKI), membrane attack complex (MAC)

## Abstract

**Background:**

Hemolytic-uremic syndrome (HUS) presents with hemolytic anemia, thrombocytopenia, and thrombotic microangiopathy of the kidney and usually results from Shiga-toxin induced activation of the alternative complement pathway. Gastroenteritis is a common feature of the Shiga-toxin producing *Escherichia coli* HUS, referred to as STEC-HUS. An inherited or acquired complement dysregulation may lead to HUS referred to as non-STEC or atypical (a)HUS. Although gastroenteritis is not a common presentation of aHUS, some patients develop ischemic colitis and may be misdiagnosed as acute appendicitis or acute ulcerative colitis (UC).

**Case Diagnosis –Treatment:**

We present a patient with low circulating complement (C) 3 levels who developed aHUS in the course of chronic active UC. Resolution of renal and gastrointestinal manifestations in response to treatment with eculizumab, a humanized monoclonal antibody against terminal C5 protein suggests the role of alternative complement in the pathogenesis of both, aHUS and UC.

**Conclusion:**

This case illustrates that dysregulation of the alternative complement pathway may manifest in other organs besides the kidney and that the circulating C3 levels do not correlate with the disease activity or the clinical response to eculizumab.

## 1. INTRODUCTION

A 16-year-old Caucasian male with a 4-year history of chronic active ulcerative colitis presented with severe symptomatic anemia, thrombocytopenia, intravascular hemolysis, acute kidney injury, nephritis, and decreased C3 levels. Three months earlier he was hospitalized for UC flare (Fig. 1a) and worsening microcytic anemia (Hb 8.2 mg/dL) and his symptoms responded to treatment with intravenous (IV) methyprednisolone and packed red blood cells (RBC) transfusion. After hospital discharge he was continued on a prednisone taper and started on 6-mercaptopurine (6MP; 1.7 mg/kg/day). One month later he developed a flu-like illness with high fever and was treated with cephalexin for possible bacterial sinusitis. Routine monitoring laboratories, including complete blood cell count and serum chemistries were normal. Over the course of the following two weeks he developed increased stool output, bloody stools, decreased appetite, fatigue, shortness of breath, and pallor. He had no urinary symptoms and no dark discoloration of urine. On arrival to the hospital he had increased blood pressure and tachycardia. Laboratory studies were remarkable for severe anemia (Hb 4.4 mg/dL), neutropenia (absolute neutrophil count 0.615 × 10^9^/L), decrease in baseline platelet count to 143 × 10^5^/mL, doubling of serum creatinine from baseline (1.4 mg/dL), increased LDH (428 IU/L), hypoalbuminemia (2.7 mg/dL), and elevated inflammatory markers (CRP 3.7 mg/dl, ESR 113 mm/h). Urinalysis revealed microscopic hematuria, 300 mg/dL protein and no pyuria. He was transfused with packed RBCs, started on IV fluids and methyprednisolone, and 6MP was discontinued. His clinical symptoms improved; however, he continued to have frequent Hemoccult positive stools. Evaluation of the peripheral blood smear, worsening thrombocytopenia (the lowest count was 38 × 10^5^/ml), rising LDH, decreased haptoglobin, and elevated free plasma hemoglobin were consistent with intravascular hemolysis (Table 1). Serum creatinine remained elevated despite hydration, and two 24-hour urine collections confirmed nephrotic range proteinuria. Serial review of urine microscopy showed interval development of RBC casts. Circulating complement (C) 3 was decreased and C4 was normal (Table 1). Stool studies were negative for *E. coli* O157:H7 by sorbitol-MacConkey agar and Shiga toxin I and II by PCR. Other workup was unremarkable except for transiently elevated homocysteine levels (Table 1).

Native kidney biopsy showed widespread and acute thrombotic microangiopathy (TMA) without chronic changes (Fig. 1b & c). Immunostaining for Membrane Attack Complex (MAC; C5b-9 complement) showed heavy granular deposition along the glomerular basement membranes of most glomeruli, with some mesangial and afferent arteriolar staining and staining of the tubular basement membrane (Fig. 1d). A working diagnosis of atypical HUS was made. After confirmation of immunization with the pneumococcal and meningococcal series, the patient received the first dose of eculizumab (900 mg). Penicillin was started for prophylaxis against *Neisseria meningitidis* serotype B. Laboratory studies did not reveal the underlying cause of aHUS (Table 1). Soluble MAC and anti-CFH antibodies were not detected before starting eculizumab, genetic studies on Complement Factor (CF)H, CFI, CFB, CFHR1-3, MCP, C3 and thrombomodulin did not reveal disease causing mutations.

**Fig. 2:**
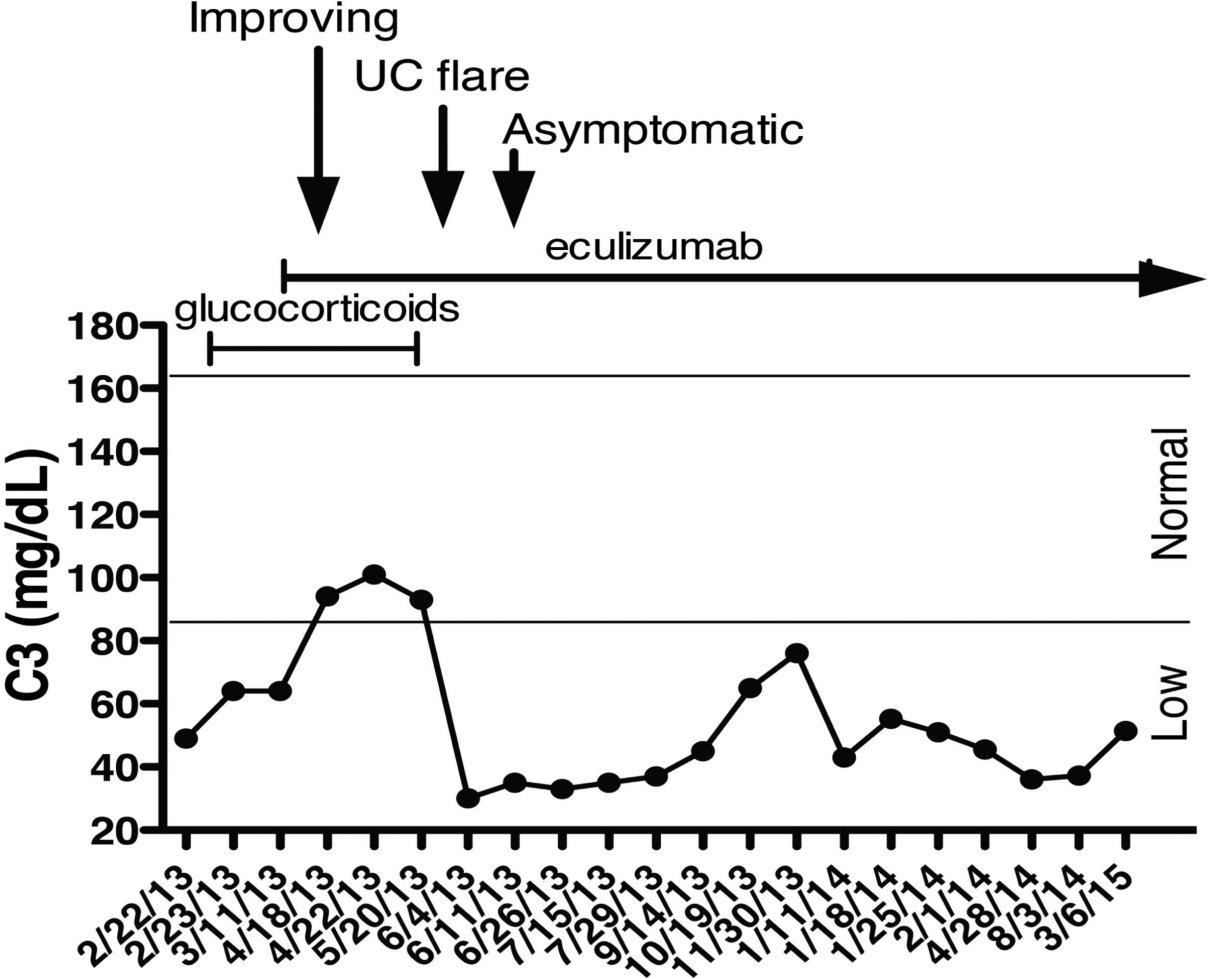
Fluctuating levels of circulating complement (C) 3. The horizontal lines delineate the normal C3 levels. Glucocorticosteroids were started on 2/23/13 and were tapered off by 5/7/13. Eculizumab was started on 3/5/13. Additional medications were metronidazole, balsalazide, penicillin, iron, and multivitamin. The patient started to improve within days after starting eculizumab but experienced a short UC flare on 5/20/13. He became asymptomatic within a week without additional therapy. The patient has been asymptomatic, normotensive and has had normal kidney function during the 24 month follow-up while remaining on eculizumab

He was discharged home on prednisone, metronidazole, balsalazide, penicillin, iron, and multivitamin. He received three additional weekly doses of eculizumab (900 mg) followed by every other week maintenance (1,200 mg). Clinical symptoms continued to improve. All blood counts and markers of inflammation and hemolysis normalized. C3 normalized briefly but subsequently remained low (Fig. 2).

Proteinuria resolved and serum creatinine returned to baseline. RBC casts were no longer present and microscopic hematuria resolved. Six weeks after initiation of eculizumab the patient experienced one brief UC flare demonstrated by increased stool output, bloody stools and elevated calprotectin (1,661.9 mcg/g) without evidence of hemolysis or AKI. The gastrointestinal symptoms resolved and calprotectin decreased to normal (<160 mcg/g) within a week with no additional therapy. Subsequently, the patient has been asymptomatic and normotensive despite having persistently low C3 levels while remaining on eculizumab. He was able to resume full activity including school attendance and participation in sports.

## 2. DISCUSSION

aHUS represents 5-10% of HUS in children but the majority of HUS in adults [1]. While the alternative complement gene mutations predispose to aHUS additional triggers are necessary for the devastating disease manifestations including platelet activation, endothelial damage, and microthrombi formation known as TMA [1]. The triggers that account for the episodic nature of aHUS are poorly understood [2,3]. Activation of the alternative complement can be caused by infectious agents, malignancy, bone marrow or solid organ transplantation and chemotherapeutic or immunosuppressive agents [4,5], systemic illness (systemic lupus erythematous, membranoproliferative glomerulonephritis, antiphospholipid antibody syndrome, scleroderma) [6,7], severe pre-ecclampsia [8], hyperhomocystinemia [9,10], mutations in the thrombomodulin (THMB) [11] or diacylglycerol kinase ε (DGKE) gene [12]. In some of these disorders TMA may result from factors outside of the complement system such as thrombophilia [9-12]. The preceding flu-like illness in the presented patient may have precipitated aHUS. By contrast, 6MP has no reported association with aHUS. Hyperhomocystinemia is an independent risk factor for intravascular thrombosis [13] and for mucosal microvascular activation in inflammatory bowel disease (IBD), including UC [14]. Many patients with IBD have elevated homocystine levels due to nutritional deficiencies or genetic polymorphisms of enzymes linked to homocysteine metabolisms [2,3]. Thus, elevated homocysteine may have been a trigger or a modifier of aHUS. Alternatively, it may have been the result of aHUS because homocysteine levels are increased in patients with renal failure [15]. The presented patient is heterozygous for the thermolabile variant 677C>T of methylenetetrahydrofolate reductase (MTHFR) necessary for re-methylation of homocysteine to methionine. However, only a homozygous or compound heterozygous mutation in the *MTHFR* gene was shown to increase homocysteine level. Further genetic testing was not performed due to normalization of the homocysteine level (7.4 μmol/L). Overall, the genetic factors leading to pathologic activation of the alternative complement cascade remain unknown in almost 50% of aHUS patients [16,17]. The specific complement gene mutation was not identified in the patient.

Historically, the clinical outcomes of aHUS have been unfavorable with up to 25% of patients dying during the acute phase and up to 50% of survivors progressing to end-stage renal disease (ESRD) [1]. The outcomes are likely to improve thanks to humanized monoclonal antibody eculizumab that blocks C5 cleavage and the formation of the pro-thrombotic, pro-inflammatory, lytic and cytotoxic terminal complement products C5a and MAC [18,19]. The patient experienced complete resolution of clinical signs and symptoms associated with TMA of the kidney and had no evidence of chronic kidney disease during 24 months of eculizumab therapy.

At least 20% of patients with primary aHUS experience extra-renal manifestations and approximately 5% of patients develop life-threatening multivisceral failure [5,20]. HUS may present with ischemic colitis and may be misdiagnosed as acute appendicitis or acute UC [21,22]. The present case is unique in that the patient developed aHUS in the course of chronic active UC. Hyperactivity of the alternative complement cascade plays a role in the pathogenesis of IBD, including UC [23-26]. It is still unclear whether deposition of C3a, C5a or MAC is responsible for inflammation in the intestinal mucosa in all IBD patients or only in a selected group. We were unable to demonstrate deposition of MAC using paraffin-embedded rectal tissue obtained during the UC flare 3 months before the diagnosis of aHUS. This procedure is less sensitive than immunostaining on frozen tissue and the negative findings in the colon may represent false negatives due to the low sensitivity for small amounts of deposit. Improvement of gastrointestinal manifestations after starting eculizumab in the patient who previously suffered severe gastrointestinal symptoms suggests the role of alternative complement pathway in the intestinal inflammation.

Similar to genetically mediated C5 deficiency, functional C5 deficiency created by eculizumab increases the risk of *Neisseria meningitidis* infection [18]. At the time of presentation, available vaccines did not cover all *Neisseria meningitidis* strains, including the most prevalent in the US serotype B. The two cases of meningococcal sepsis reported during eculizumab exposure occurred in immunized patients who were not receiving antibiotic prophylaxis [27]. The patient has continued antibiotic prophylaxis for *Neisseria meningitidis* without experiencing infectious complications 24 months after the initiation of therapy.

Deposition of C3 and MAC in the endothelium can predict complement activation in aHUS [28]. While circulating C3 levels are decreased in 30-50% of aHUS patients [1,29], the levels do not correlate with the disease activity or the clinical response to eculizumab [28]. This is illustrated by the fluctuating C3 levels in the patient (Fig. 2). Specific and sensitive markers of complement activation in aHUS are lacking and such markers are needed for the diagnostic and prognostic evaluation and for monitoring the C5 blockade in response to eculizumab.

## 3. CONCLUSION

Dysregulation of the alternative complement pathway may not only manifest in the kidney but other organs may be involved as well. As illustrated by this case, there is no correlation between C3 levels and disease activity in aHUS, but resolution of clinical symptoms is important to establish effectiveness of therapy. Biomarkers of complement activation in aHUS are needed for the diagnostic and prognostic evaluation and for monitoring the C5 blockade in response to eculizumab.

## Figures and Tables

**Fig. 1. (a):**
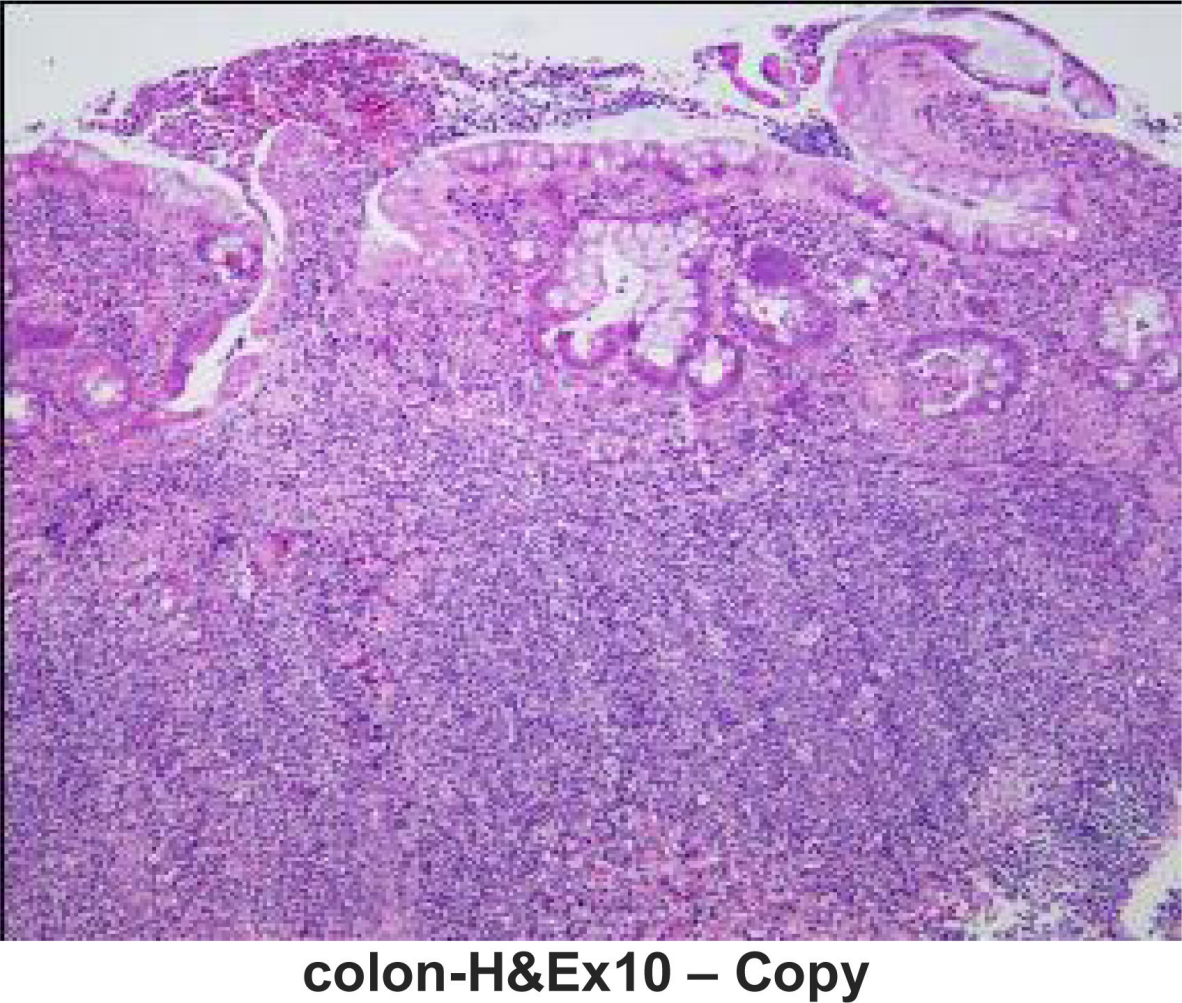
Light microscopy of colon biopsy specimen demonstrating chronic active colitis. Thickened muscularis mucosae, lamina propria fibrosis, crypt distortion, and heavy lymphoid and plasma cell component as well as stromal neutrophils and crypt abscess formation (arrow). (Hematoxylin and eosin; original magnification × 100). Specimen was obtained 3 months before presentation with aHUS

**Fig. 1. (b):**
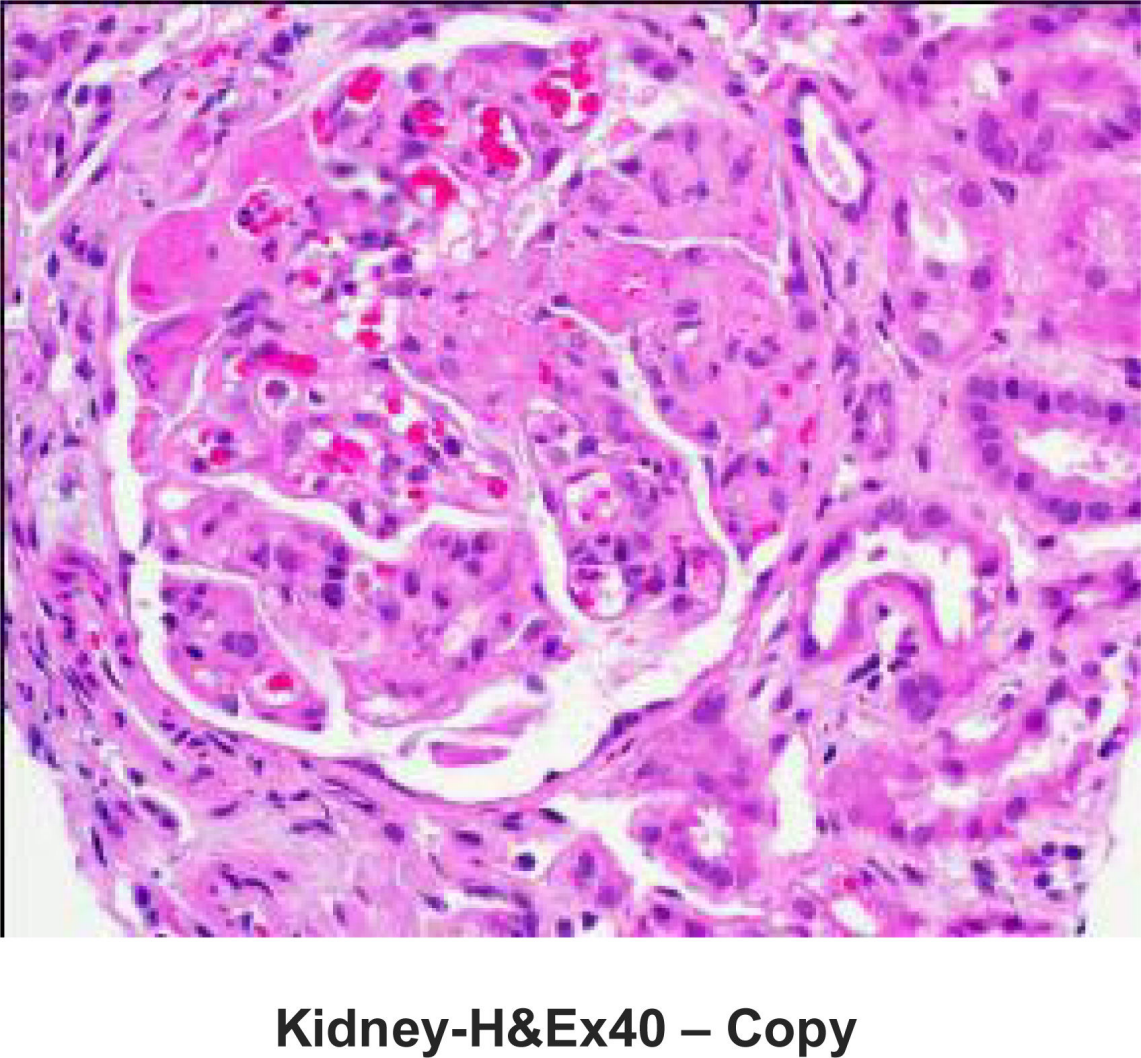
Light microscopy of kidney biopsy specimen demonstrating acute thrombotic microangiopathy without chronic findings of glomerulosclerosis or interstitial fibrosis. There is glomerular capillary dilatation, increased mesangial matrix, as well as platelet and fibrin thrombi in glomerular loops with focal and segmental necrosis. There is extension to afferent arterioles with fibrinoid change. RBC fragments are present in the glomerulus. Tubules have large resorption droplets, are often dilated with RBC casts. The interstitium has focal RBC (Hematoxylin and eosin; original magnification × 400)

**Fig. 1. (c):**
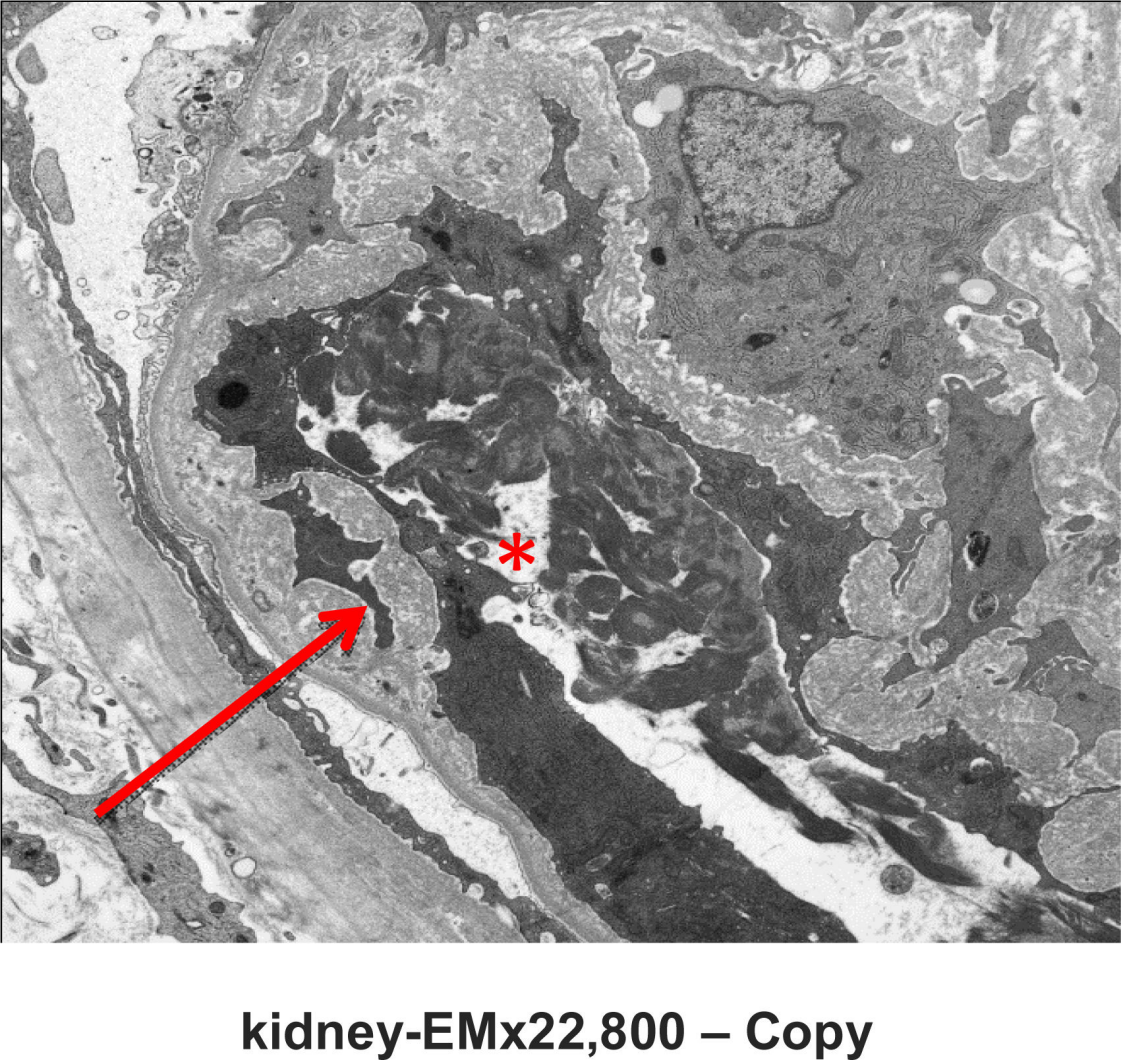
Electron microscopy of the glomerular capillary loop. Fibrin is present in a capillary lumen (asterisk) and there is fibrillar material that separates the endothelial cell from the capillary basement membrane (arrow) (Original magnification × 22,800)

**Fig. 1. (d):**
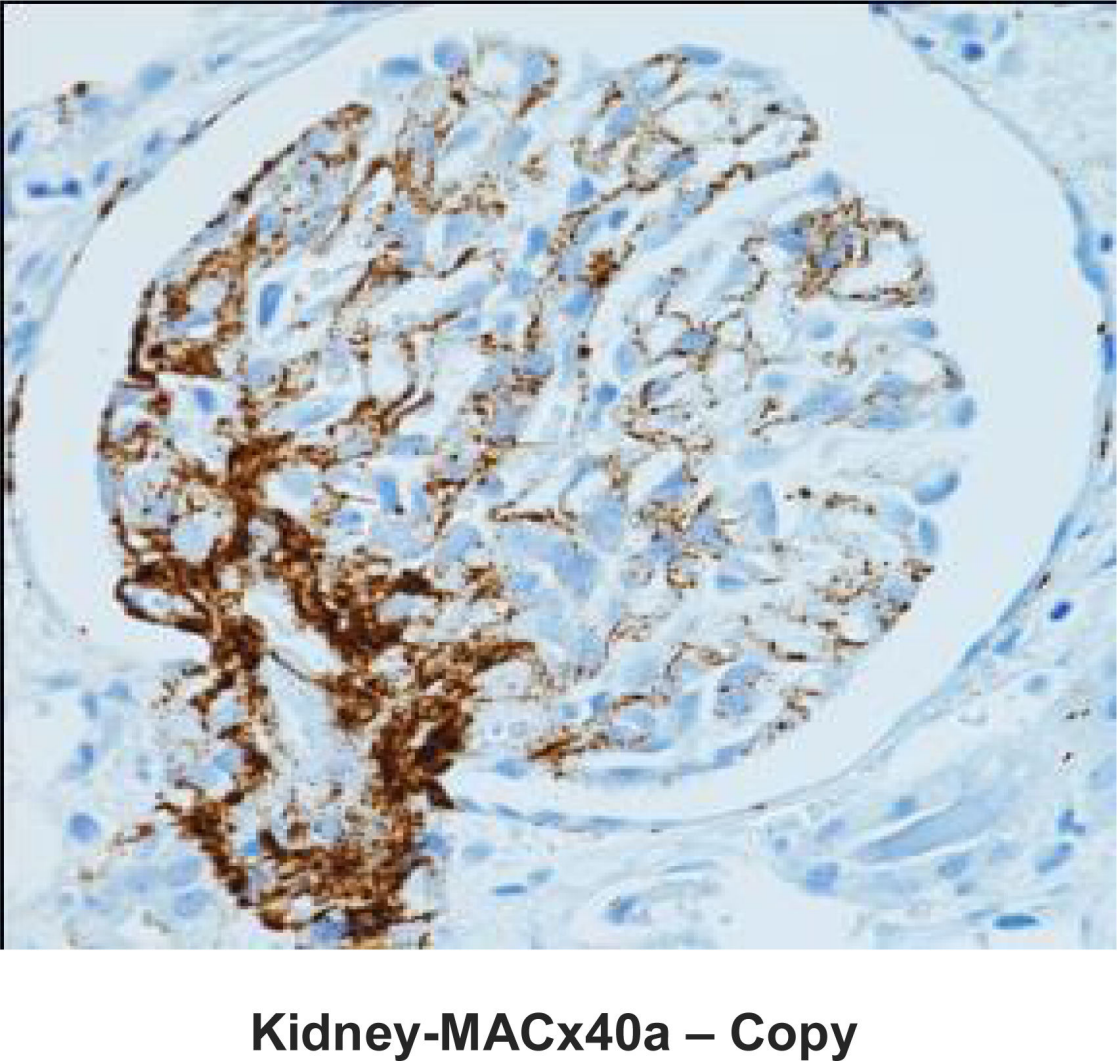
Immunostaining (antibody DAKO #M0777, clone aE11) for membrane attack complex (MAC; C5b-9 complement) of kidney biopsy specimen demonstrating heavy granular deposition along the glomerular basement membranes with some mesangial and afferent arteriolar staining. (Original magnification × 400)

**Table 1:** The laboratory test results

Direct coombs	Negative
Indirect coombs	Negative
Haptoglobin	<15 mg/dL (30-200)
LDH	428-735 IU/L (115-257)
Free plasma hemoglobin	11.7 (<6)
ESR	113 mm/h (< 20)
CRP	3.7 mg/dL (0.04-0.79)
C3	49 mg/dL (82-163)
C4	18 mg/dL (14-41)
PNH flow cytometry	Negative
ADAMTS13 activity	85% (>68)
ADAMTS13 inhibitor	15% (<30)
Ristocetin cofactor	1.77 U/mL(0.5-1.5)
Russel viper venom time	Normal
APC resistance	3.4 (2.1-3)
PT/PTT/INR	Normal
Thrombin	Normal
Protein C	Normal
Protein S	170% (58-128)
Factor X	Normal
Antithrombin III	Normal
Homocysteine	19.8 umol/L (5.5-13.8)
MTHFR gene	677C>T variant, heterozygous
Prothrombin gene	No 20210G>A mutation
ANA	Negative
ANCA	Negative
LAC/vWD	Negative
ASO titer	Negative
anti-dNAse B antibody	Negative
Anti-dsDNA antibody	Negative
CFH level	256 mcg/ml (160-412)
Anti-CFH antibody	Negative
CFH coding region	No disease causing mutations
MLPA for CFHR3-1 deletion	Negative
CFB level	205.6 mcg/ml (127.6-278.5)
CFB coding region	No disease causing mutations
CFI level	44.0 mcg/ml (29.3-58.5)
CFI coding region	No disease causing mutations
MCP	Normal
MCP coding region	No disease causing mutations
ELISA for TCC	Negative (no TCC consumption)
C3 coding region	No disease causing mutations
Thrombomodulin gene	No disease causing mutations

## ABBREVIATIONS

LDH: lactate dehydrohenase
ESR: erythrocyte sedimentation rate
CRP: C-reactive protein
C: complement protein
PNH: paroxysmal nocturnal hemoglobinuria
ADAMTS: a disintegrin and metalloproteinase with a thrombospondin type 1 motif
APC: activated Protein C
MTHFR: methylenetetrahydrofolate reductase
ANA: anti-nuclear antibody
ANCA: antineutrophil cytoplasmic antibody
ASO: anti-streptolysin O; methylenetetrahydrofolate reductase
LAC: lupus anticoagulant
vWD: vonWillebrand factor
CF: complement factor
dsDNA: double stranded DNA
MPLA: multiplex ligation-dependent probe amplification testing
MCP: membrane cofactor protein
TCC: terminal complement complex; Normal values are in parenthesis

## References

[1] Loirat C, Fremeaux-Bacchi V (2011). Atypical hemolytic uremic syndrome.. Orphanet J Rare Dis.

[2] Romagnuolo J, Fedorak RN, Dias VC, Bamforth F, Teltscher M (2001). Hyperhomocysteinemia and inflammatory bowel disease: Prevalence and predictors in a cross-sectional study.. Am J Gastroenterol.

[3] Jiang Y, Xia X, Wang W (2012). Hyperhomocysteinemia and related genetic polymorphisms correlate with ulcerative colitis in Chinese Han population in Central China [corrected].. Cell Biochem Biophys.

[4] Loomis LJ, Aronson AJ, Rudinsky R, Spargo BH (1989). Hemolytic uremic syndrome following bone marrow transplantation: a case report and review of the literature.. Am J Kidney Dis.

[5] Komeno Y, Ogawa S, Ishida T (2003). Ischemic colitis as a manifestation of thrombotic microangiopathy following bone marrow transplantation.. Intern Med.

[6] Lansigan F, Isufi I, Tagoe CE (2011). Microangiopathic haemolytic anaemia resembling thrombotic thrombocytopenic purpura in systemic lupus erythematosus: the role of ADAMTS13.. Rheumatology (Oxford).

[7] Skerka C, Licht C, Mengel M (2009). Autoimmune forms of thrombotic microangiopathy and membrano-proliferative glomerulonephritis: Indications for a disease spectrum and common pathogenic principles.. Mol Immunol.

[8] Ganesan C, Maynard SE (2011). Acute kidney injury in pregnancy: The thrombotic microangiopathies.. J Nephrol.

[9] Geraghty MT, Perlman EJ, Martin LS (1992). Cobalamin C defect associated with hemolytic-uremic syndrome.. J Pediatr.

[10] Van Hove JL, Van Damme-Lombaerts R, Grunewald S (2002). Cobalamin disorder Cbl-C presenting with late-onset thrombotic microangiopathy.. Am J Med Genet.

[11] Delvaeye M, Noris M, De Vriese A (2009). Thrombomodulin mutations in atypical hemolytic-uremic syndrome.. N Engl J Med.

[12] Lemaire M, Fremeaux-Bacchi V, Schaefer F (2013). Recessive mutations in DGKE cause atypical hemolytic-uremic syndrome.. Nat Genet.

[13] Kemp EJ, Strain L, Diaz-Torres ML, Goodship JA, Goodship TH (2005). The development of atypical hemolytic uremic syndrome is not influenced by thrombophilia susceptibility factors.. J Thromb Haemost.

[14] Danese S, Sgambato A, Papa A (2005). Homocysteine triggers mucosal microvascular activation in inflammatory bowel disease.. Am J Gastroenterol.

[15] van Guldener C (2006). Why is homocysteine elevated in renal failure and what can be expected from homocysteine-lowering?. Nephrol Dial Transplant.

[16] Noris M, Remuzzi G (2009). Atypical hemolytic-uremic syndrome.. N Engl J Med.

[17] Noris M, Mescia F, Remuzzi G (2012). STECHUS, atypical HUS and TTP are all diseases of complement activation.. Nat Rev Nephrol.

[18] Zuber J, Fakhouri F, Roumenina LT, Loirat C, Fremeaux-Bacchi V, French Study Group for a HCG (2012). Use of eculizumab for atypical haemolytic uraemic syndrome and C3 glomerulopathies.. Nat Rev Nephrol.

[19] Legendre CM, Licht C, Muus P (2013). Terminal complement inhibitor eculizumab in atypical hemolytic-uremic syndrome.. N Engl J Med.

[20] Fremeaux-Bacchi V, Fakhouri F, Roumenina L, Dragon-Durey MA, Loirat C (2011). [Atypical hemolytic-uremic syndrome related to abnormalities within the complement system].. Rev Med Interne.

[21] Craner GE, Burdick GE (1976). Acute colitis resembling ulcerative colitis in the hemolytic-uremic syndrome.. Am J Dig Dis.

[22] Dillard RP (1983). Hemolytic-uremic syndrome mimicking ulcerative colitis. Lack of early diagnostic laboratory findings.. Clin Pediatr (Phila).

[23] Johswich K, Martin M, Bleich A (2009). Role of the C5a receptor (C5aR) in acute and chronic dextran sulfate-induced models of inflammatory bowel disease.. Inflamm Bowel Dis.

[24] Sugihara T, Kobori A, Imaeda H (2010). The increased mucosal mRNA expressions of complement C3 and interleukin-17 in inflammatory bowel disease.. Clin Exp Immunol.

[25] Lu F, Fernandes SM, Davis AE (2010). The role of the complement and contact systems in the dextran sulfate sodium-induced colitis model: the effect of C1 inhibitor in inflammatory bowel disease.. Am J Physiol Gastrointest Liver Physiol.

[26] Jain U, Woodruff TM, Stadnyk AW (2013). The C5a receptor antagonist PMX205 ameliorates experimentally induced colitis associated with increased IL-4 and IL-10.. Br J Pharmacol.

[27] Hillmen P, Muus P, Roth A (2013). Long-term safety and efficacy of sustained eculizumab treatment in patients with paroxysmal nocturnal haemoglobinuria.. Br J Haematol.

[28] Noris M, Galbusera M, Gastoldi S (2014). Dynamics of complement activation in atypical HUS and how to monitor eculizumab therapy.. Blood.

[29] Noris M, Caprioli J, Bresin E (2010). Relative role of genetic complement abnormalities in sporadic and familial aHUS and their impact on clinical phenotype.. Clin J Am Soc Nephrol.

